# Neuroimaging studies of cannabidiol and potential neurobiological mechanisms relevant for alcohol use disorders: a systematic review

**DOI:** 10.1186/s42238-024-00224-0

**Published:** 2024-03-21

**Authors:** Tristan Hurzeler, Joshua Watt, Warren Logge, Ellen Towers, Anastasia Suraev, Nicholas Lintzeris, Paul Haber, Kirsten C. Morley

**Affiliations:** 1 Faculty of Medicine and Health, University of Sydney, Sydney, NSW Australia; 2 Translational Research in Alcohol, Edith Collins Centre, Sydney Local Health District, Sydney, Australia; 3 Lambert Initiative for Cannabinoid Therapeutics, University of Sydney, Sydney, NSW Australia; 4 Drug and Alcohol Services, South Eastern Sydney Local Health District, Sydney, Australia

**Keywords:** Cannabidiol, Neuroimaging, PET, Alcohol use disorder, Pharmacotherapy

## Abstract

The underlying neurobiological mechanisms of cannabidiol’s (CBD) management of alcohol use disorder (AUD) remains elusive.

**Aim** We conducted a systematic review of neuroimaging literature investigating the effects of CBD on the brain in healthy participants. We then theorise the potential neurobiological mechanisms by which CBD may ameliorate various symptoms of AUD.

**Methods **This review was conducted according to the PRISMA guidelines. Terms relating to CBD and neuroimaging were used to search original clinical research published in peer-reviewed journals.

**Results **Of 767 studies identified by our search strategy, 16 studies satisfied our eligibility criteria. The results suggest that CBD modulates γ-Aminobutyric acid and glutamate signaling in the basal ganglia and dorso-medial prefrontal cortex. Furthermore, CBD regulates activity in regions associated with mesocorticolimbic reward pathways; salience, limbic and fronto-striatal networks which are implicated in reward anticipation; emotion regulation; salience processing; and executive functioning.

**Conclusion **CBD appears to modulate neurotransmitter systems and functional connections in brain regions implicated in AUD, suggesting CBD may be used to manage AUD symptomatology.

## Introduction

The medical, psychological, and social sequelae of alcohol use disorder (AUD) are major global public health concerns. Harmful alcohol consumption is linked to many physical and mental health complications and is responsible for 5.1% of the global burden of disease (Griswold et al. 2018; WHO 2018, 2021). AUD, particularly when moderate to severe, is a chronic relapsing disorder, characterized by compulsive alcohol-seeking and consumption despite negative repercussions to both physical and mental health (Haber, Riordan, & Morley 2021). A wealth of research suggests that neurobiological changes to various neurotransmitter systems and brain circuits underpin the behaviour and psychology which maintains AUD (Koob & Volkow 2016). Primary neurotransmitter systems influenced by prolonged and heavy alcohol consumption include dopaminergic, γ-aminobutyric acidergic (GABA)-ergic, glutamatergic, serotonergic, and opioidergic (Chastain 2006; Vitale, Iannotti, & Amodeo 2021). Pharmacotherapy can be useful, in conjunction with psychosocial support, for reducing the core symptoms of AUD (such as reducing craving, habitual seeking behaviours, and withdrawal) and achieving abstinence or aiding the control of consumption (Morley et al. 2021). However, there currently exists a paucity of medications available to treat AUD (Morley 2021).

Neuroimaging literature has identified specific neurocircuit and biochemical alterations thought to be responsible for the observed cognitive and behavioural changes associated with AUD. Changes to mesocorticolimbic reward pathways, following steep increases in opioid and D1 signaling into the ventral striatum, leads to increases in reward anticipation and salience attribution to drug-related cues which leads to increased drug-seeking behaviours (Koob & Volkow 2016). Further, reduced signaling of dopaminergic systems in reward and limbic networks leads to negative emotion, anhedonia, and heightened stress (Koob & Volkow 2016). Finally, fronto-striatal network and fronto-cortical dysregulation leads to reduced executive functioning and emotion regulation (Jentsch & Taylor 1999). Understanding the brain correlates of AUD and implementing neuroimaging techniques to identify the methods by which novel pharmacotherapies may modulate these correlates provide a method for more effective and tailored treatments.

Over the past few years there has been an influx of research exploring CBD as a potential pharmacotherapy for a variety of indications due to its wide-ranging therapeutic effects and favourable safety profile (José A Crippa et al. 2018). CBD is the second most abundant chemical constituent of the *Cannabis sativa* plant and, unlike Δ9-tetrahydrocannabinol (THC), is non-intoxicating and has nil potential for abuse or dependence (Arout, Haney, Herrmann, Bedi, & Cooper, 2022; Bergamaschi et al. 2011; Haney et al. 2016; Leweke et al. 2012; McCartney et al. 2022; Schoedel et al. 2018). CBD has shown to possess affinity for multiple targets including the modulation of serotoninergic, dopaminergic, glutamatergic, GABAergic (Scopinho et al. 2011) and endocannabinoid signaling (Corroon, Felice, & Medicine 2019). This multi-target action of CBD may explain the various therapeutic properties including antiepileptic (Devinsky et al. 2016; Talwar, Estes, Aparasu, & Reddy, 2022), anxiolytic (Berger et al. 2022; Bhattacharyya & et al. 2018; Stefan J. Borgwardt et al. 2008; Paolo Fusar-Poli et al. 2010; Jadoon, Tan, & O’Sullivan 2017; Wilson et al. 2019a), neuroprotective (José A Crippa et al. 2018), and anti-inflammatory and antioxidant effects (Mandolini et al. 2018; Mechoulam et al. 2007; Ren et al. 2009). This combination of potential therapeutic effects suggests that CBD might be particularly well suited to management of alcohol use disorder. In fact, CBD may modulate drug craving and seeking behaviours. CBD has been shown to reduce craving and anxiety in heroin users (Hurd et al. 2019), as well as stress and drug cue alcohol reinstatement, voluntary alcohol consumption, withdrawal symptoms and alcohol induced relapse behaviours in preclinical models of alcohol dependence (Viudez-Martínez et al. 2018a, b, c; A. Viudez-Martínez et al. 2018a, b, c ). This suggests that CBD could protect from further damage of alcohol due to its neuroprotective and anti-oxidant properties which could improve executive functioning, but may also modulate key disorder characteristics which precipitate relapse such as heightened anxiety (Skelley et al. 2020) and craving in response to alcohol cues and stressors (Hurd et al. 2019). Neuroimaging techniques provide valuable insights into the structure and function of the brain and may explain the relationship between the pharmacological action of CBD and its behavioural and psychological effects (Hargreaves et al. 2015; Nathan et al. 2014; Wong et al. 2009). However, there has currently been no attempt to compile and compare neuroimaging studies to examine whether the converging neurobiological effects of CBD are relevant to AUD. To establish the current understanding of the neurobehavioral mechanisms of action of CBD on the human brain, and its pharmacotherapeutic potential for AUD, we examined common neuroimaging techniques including, magnetic resonance spectroscopy (MRS), magnetic resonance imaging (MRI, including both functional and structural imaging), single photon emission computed tomography (SPECT) and positron emission tomography (PET). MRI is a non-invasive technique that produces anatomical images of the brain used to investigate both structural and functional aspects of the brain. Structural MRI provides a snapshot of brain anatomy in time while functional MRI (fMRI) can identify brain activity occurring during a variety of cognitive and functional activities of the brain in real-time. Specific cognitive phenomena can be targeted by presenting participants with specific tasks, known as task fMRI (tfMRI) (Heeger & Ress 2002; Linden et al. 1999; Worsley & Friston 1995) or also conducted in task-free paradigms known as resting state fMRI (rsfMRI) (Fox & Raichle 2007; Raichle et al. 2001). Magnetic resonance spectroscopy (MRS) is an imaging modality that can identify the presence and density of a variety of neurometabolites in the brain. Finally, nuclear imaging techniques PET and SPECT use radiotracers which are absorbed by the body and the resulting emission of positrons (in the case of PET) and gamma rays (in the case of SPECT) provides a measure of cellular and molecular function. The destination of the radiotracers indicates the location of changes in metabolic and other physiological processes such as blood flow, and regional chemical absorption.

This review aimed to systematically examine studies using these imaging techniques to elucidate the neurobehavioural and neuropsychological effects of CBD, as well as provide insights into the potential mechanism of CBD in the management of key symptoms of AUD.

## Methods

This review follows the Preferred Reporting Items for Systematic Reviews and Meta-analyses (PRISMA) guidelines for systematic reviews (Moher et al. 2009). Prior to the commencement of the data extraction, this review was registered with the international prospective register of systematic reviews (PROSPERO # CRD42021272561). The original protocol can be accessed on the PROSPERO website.

### Search strategy

Terms relating to CBD, and neuroimaging were used to search EMBASE, PubMed, Medline and PsycINFO databases. This search strategy used a combination of MeSH heading and key words and used two main sections. These sections related to “cannabidiol” and a section relating to imaging techniques i.e. (MRS OR Magnetic Resonance Spectroscopy OR Spectroscopy OR Metabolite Concentrations OR magnetic resonance spectroscopy OR MRS OR functional magnetic resonance imaging OR fMRI OR resting state functional OR magnetic resonance imaging OR rsfMRI OR structural magnetic resonance imaging OR MRI OR magnetic resonance spectroscopy OR PET OR positron emission tomography). The search was re-run in June 2022 to capture any new publications.

### Study selection

Once all the database searches had been completed and duplicate studies removed, a multi-stage screening process was performed by one author (TH). Studies were screened in the following order i) title ii) abstract iii) full-text article. Titles were screened to ensure studies used CBD as the active medication and that neuroimaging outcomes were the key measure of interest. Abstracts were then further assessed to ensure only human studies were included. In the final stage, all remaining studies had a full-length text review to ensure that the study satisfied more specific inclusion criteria.

### Eligibility criteria

Studies which investigated the effect of CBD on the brain using either MRI, fMRI, MRS, SPECT or PET in human subjects were included. All eligible studies also had to include an experimental group that received CBD which did not fit diagnostic criteria for a mental health disorder. Studies were excluded if they were post-mortem, animal investigations, non-brain MRI studies, or examined the effect of cannabis rather than CBD. Review and non-English articles were excluded. The reference list of all eligible studies was also manually searched to identify any additional publications.

### Data extraction

The following data was extracted from all eligible studies: author, year of publication, number of participants in patient and control groups, age, proportion of males and females, clinical condition and diagnosis (patients only), matching factors in controls, neuroimaging paradigm, scanner specifics, outcome variables including: i) structural brain changes; ii) CBD-modulated brain activity (as measured through the blood oxygen level dependent [BOLD] response) or functional connectivity; iii) CBD-induced alterations in metabolites such as glutamate, GABA, and glutamine; iv) CBD-induced alterations in metabolism of blood PET.

### Quality assessment

Risk of bias was assessed by the AXIS for cross-sectional studies or the Cochrane Risk of Bias (Sterne et al. 2019) for randomised trials with both crossover and parallel designs. The risk of bias assessment was assessed independently by two authors (KM and JW) and any discrepancies were resolved by discussion between the two authors with consultation available from a third party if required.

## Results

### General overview of study selection process

**Fig. 1:**
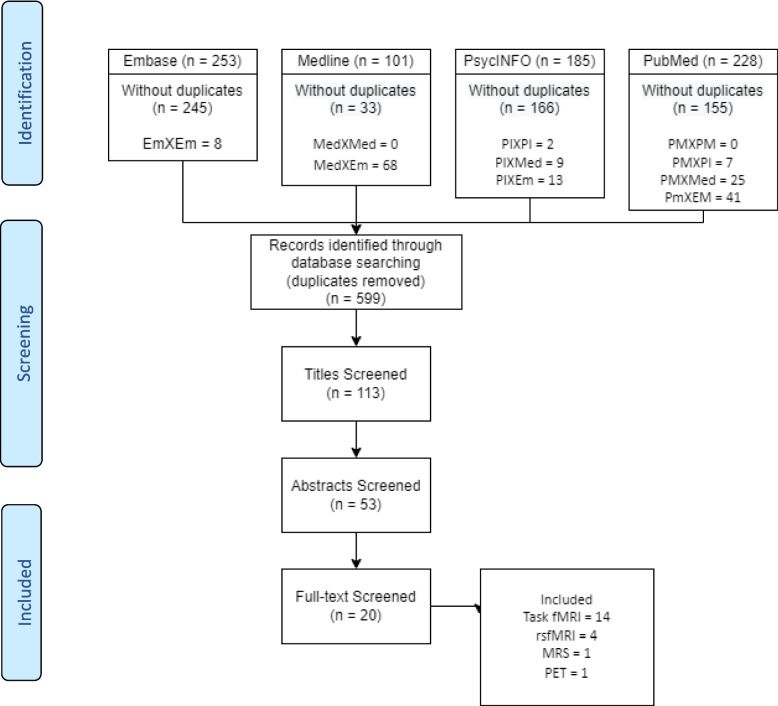
PRISMA flow chart. Preferred Reporting Items for Systematic Reviews and Meta-analyses (PRISMA) flow diagram of the study selection process. EM = Embase; Med = Medline; PI = PsycINFO; PM = PubMed; MRI = magnetic resonance imaging; fMRI = functional MRI; rsfMRI = resting state fMRI; MRS = magnetic resonance spectroscopy; PET = positron emission tomography

**Fig. 2:**
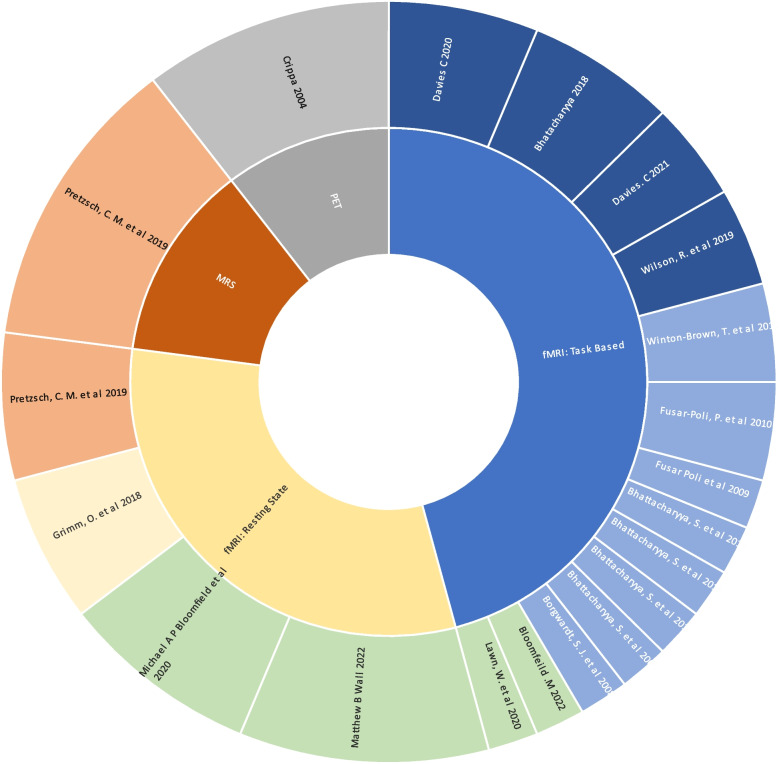
Sunburst chart. The proportion of studies using each neuroimaging modality in the inner ring. Each colour in the outer ring indicates a different participant sample

The primary search identified 767 records from four databases see Fig. 1 in Appendix . After removing duplicates, 599 items remained. Titles were screened first and items that did meet eligibility criteria were removed leaving 113 studies. After screening abstracts, 53 studies remained. Finally, the full-texts were screened leaving 16 studies for inclusion in the review. Before publication, a secondary search in June 2022 identified four new studies that were included in the final version of the review. The 20 included studies all administered CBD orally with 19 studies dosing 600 mg CBD and one 400 mg CBD. Fourteen of the 20 included studies were task-based fMRI, four were rsfMRI, one study was an MRS study, and one study used SPECT imaging Although search terms related to SPECT were not included in the search strategy, this study was included due to its pertinence to the focus of this review. Additionally, various studies were used the same or similar participant samples as depicted by the colour categorisations of the outer ring of the sunburst plot (Fig. 2 in the Appendix). In “Functional MRI” section we detail functional (subdivided into rsfMRI and different task paradigms) and neurochemical findings (Tables 1 and 2).

**Table 1:** Data extraction table

ID	Title	Design	Medication	Scanner details	Results (CBD only)
	fMRI: Task Based				
1a	Borgwardt, S. J. et al. 2008	DBPC pseudo-randomized, repeated measures, within-subject design. 1-month washout	THC 10 mg or CBD 600 mg or placebo	1.5 T Sigma (GE)	CBD deactivated the left temporal cortex and insula. These effects were not related to changes in anxiety, intoxication, sedation, and psychotic symptoms. Trend for less anxiety following CBD relative to placebo (*p* = 0 .06)
1b	Bhattacharyya, S. et al. 2009	DBPC pseudo-randomized, repeated measures, within-subject design. 1-month washout	THC 10 mg or CBD 600 mg or placebo	1.5 T Sigma (GE)	CBD induced trend in modulation of insula, mediotemporal gyrus, lingual gyrus, precuneus and precentral gyrus during encoding blocks and in hippocampus during recall blocks but did not survive threshold for less than 1 false positive
1c	Bhattacharyya, S. et al. 2010	DBPC pseudo-randomized, repeated measures, within-subject design. 1-month washout	THC 10 mg or CBD 600 mg or placebo	1.5 T Sigma (GE)	CBD attenuated amygdala response while observing fearful which correlated with an anxiolytic effect (*r* = 0.551, *p* = 0.017). CBD also decrease amygdala response also correlated with reductions in galvanic skin response while and intensely fearful faces (*r* = 0.524; *p* = 0.049) Right temporal cortex, parahipcampal gyrus, insula and caudate were augmented by CBD during response inhibition task While listening to speech CBD the superior temporal cortex was augmented by CBD During visual processing the occipital lobe was augmented During verbal recall CBD was associated with a trend increase in activity in the striatum compared to placebo
1d	Bhattacharyya, S. et al. 2012	DBPC pseudo-randomized, repeated measures, within-subject design. 1-month washout	THC 10 mg or CBD 600 mg or placebo	1.5 T Sigma (GE)	CBD attenuated activation in the left medial prefrontal cortex and augmented activation in the right caudate, parahippocampal gyrus, insula, precentral gyrus and thalamus, relative to placebo during oddball salience processing. CBD also reduced response latencies
1e	Bhattacharyya, S. et al. 2014	DBPC pseudo-randomized, repeated measures, within-subject design. 1-month washout	THC 10 mg or CBD 600 mg or placebo	1.5 T Sigma (GE)	CBD increased fronto-striatal connectivity but decreased mediotemporal-prefrontal connectivity during oddball salience processing
1f	Fusar Poli et al. 2009	DBPC pseudo-randomized, repeated measures, within-subject design. 1-month washout	THC 10 mg or CBD 600 mg or placebo	1.5 T Sigma (GE)	CBD attenuated the BOLD signal in the amygdala and anterior and posterior cingulate cortex while subjects were processing intensely fearful faces, and its suppression of the amygdalar and anterior cingulate response was correlated with the concurrent reduction in SCR fluctionations. CBD also reduced activity compared to placebo in the posterior lobe of the cerebellum for 50% fearful face stimuli
1 g	Fusar-Poli, P. et al. 2010	DBPC pseudo-randomized, repeated measures, within-subject design. 1-month washout	THC 10 mg or CBD 600 mg or placebo	1.5 T Sigma (GE)	In the placebo condition, BMS identified a model with driving inputs entering via the anterior cingulate and forward intrinsic connectivity between the amygdala and the anterior cingulate as the best fit. CBD but not D9-THC disrupted forward connectivity between these regions during the neural response to fearful faces
1 h	Winton-Brown, T. et al. 2011	DBPC pseudo-randomized, repeated measures, within-subject design. 1-month washout	THC 10 mg or CBD 600 mg or placebo	1.5 T Sigma (GE)	CBD had no significant symptomatic effects in anxiety, intoxication, and positive psychotic symptoms. CBD was associated with activation in right temporal cortex during auditory processing
2a	Wilson, R. et al. 2019	DBRPC parallel-arm study	CHR received 600 mg CBD or matched placebo, while HC received no treatment	GeneralElectric Signa HDx 3.0 T MRI scanner	CBD attenuated the hyperactivity in the left insula/parietal operculum for CHR participants and was associated with overall slowing of reaction time
2b	Davies. C 2022	DBRPC parallel-arm study	CHR received 600 mg CBD or matched placebo, while HC received no treatment	GeneralElectric Signa HDx 3.0 T MRI scanner	Healthy controls showed a significant negative relationship between cortisol and parahippocampal activation (*p* = 0.023). During fear processing, increases in cortisol levels induced by social stress led to lower parahippocampal activation. This relationship was significantly different in placebo compared to healthy controls (*p* = 0.033) but not CBD conditions vs healthy controls (*p* = 0.67)
2c	Bhattacharyya 2018	DBRCT, parallel-arm study	CHR received 600 mg CBD or matched placebo, while HC received no treatment	GeneralElectric Signa HDx 3.0 T MRI scanner	In the CBD group activation was greater than in the placebo group but lower than in the control group in the right caudate during encoding and in the parahippocampal gyrus and midbrain during recall. The level of activation in the CBD group was thus intermediate to that in the other 2 groups
2d	Davies C 2020	DBRCT, parallel-arm study	CHR received 600 mg CBD or matched placebo, while HC recived no treatment	GeneralElectric Signa HDx 3.0 T MRI scanner	During fear processing, CHR participants receiving CBD showed greater activation than HC but lower activation than those who received placebo in the parahippocampal gyrus. CHR participants receiving CBD showed lower activation than HC but higher activation than those who received placebo in the striatum
3a	Lawn, W. et al. 2020	DBRPC repeated measures, crossover design	600 mg oral dose of CBD and matched placebo	3-Tesla Siemens Prisma MRI Scanner	There was insufficient evidence to suggest that CBD altered reward-related brain activity
3b	Bloomfeild.M 2022	DBRPC repeated measures, crossover design	600 mg oral dose of CBD and matched placebo	3-Tesla Siemens Prisma MRI Scanner	There was insufficient evidence to suggest that CBD altered brain regions associated with emotional processing or responding to emotional faces
	fMRI: Resting State				
3c	Michael A P Bloomfield et al 2020	DBRPC repeated measures, crossover design	600 mg CBD or placebo	3-Tesla Siemens Prisma MRI Scanner	CBD increased CBF in the hippocampus (*p* = 0.004). There was no effect on memory task performance, but there was a significant correlation whereby greater CBD-induced increases in orbitofrontal CBF were associated with reduced reaction time on the 2-back working memory task ( *r* = − 0.73, *p* = 0.005)
3d	Matthew B Wall 2022	DBRPC repeated measures, crossover design	600 mg CBD or placebo	3-Tesla Siemens Prisma MRI Scanner	Compared to placebo, CBD was associated with a relative increase between areas in the posterior parietal lobes, parietooccipital sulcus, the left posterior cingulate and areas of the striatum involved in association CBD also led to decreased connectivity was found in the right hemisphere insula and lateral frontal cortex. Furthermore, CBD relatively decreased connectivity from the striatum sensorimotor seed-region and left cerebellum
4	Grimm, O. et al. 2018	Subject observer- blinded, RCT Crossover	10 mg THC vs CBD 600 mg vs placebo	3-Tesla Siemens Trio	Increase in front striatal coupling during intake of 600 mg CBD. ROI-putamen showed increased activity with three clusters in the frontal lobe
5a	Pretzsch, C. M. et al. 2019	DBRPC, repeated-measures, cross-over study	600 mg CBD or placebo	3 T GE Excite II	Primarily driven by the ASD group, with no significant change in controls, CBD significantly increased fALFF in the right fusiform gyrus (*p* = 0.041) and in the cerebellar vermis VI (*p* = 0.048). Within the ASD group only, CBD also significantly altered vermal functional connectivity with several of its subcortical (striatal) and cortical targets
	MRS				
5b	Pretzsch, C. M. et al. 2019	DBRPC, repeated-measures, cross-over study	600 mg CBD or placebo	3 T GE Excite II	Across regions, CBD increased GABA + in controls, but decreased GABA + in ASD; the group difference in change in GABA + in the DMPFC was significant
	PET				
6	Crippa J. et al. 2004	DBRCT	400 mg CBD	Double-detector SOPHAs DST system	CBD was associated with an increased parahippocampal gyrus blood flow. CBD conditions also showed decreased blood flow to a mediotemporal cluster including the left amygdala-hippocampal complex, hypothalamus, and a cluster in the left posterior cingulate gyrus blood flow

*ASD* autism spectrum disorder, *CBD* cannabidiol, *DBRPC*, double-blind, randomized, placebo-controlled, *DBRCT* Double blind, randomised control trial, *MIDT* monetary incentive delay task, *ROI* Region of interest, *fLAFF* ‘fractional amplitude of low-frequency fluctuations’, *GABA* γ-aminobutyric acidergic

**Table 2:** Sample demographics

Sample ID	Name	Participants	Age (mean [sd])	Sex (female%)	Recruited
	fMRI: Task Based				
1a	Borgwardt, S. J. et al. 2008	15 healthy men	26.7 [5.7]	0%	Recruited through advertisement in the local media
1b	Bhattacharyya, S. et al. 2009	15 healthy men	26.67 [5.7]	0%	Recruited through advertisement in the local media
1c	Bhattacharyya, S. et al. 2010	15 healthy men	26.7 [5.7]	0%	Recruited through advertisement in the local media
1d	Bhattacharyya, S. et al. 2012	15 healthy men	26.67 [5.7]	0%	Recruited through advertisement in the local media
1e	Bhattacharyya, S. et al. 2014	15 healthy men	26.67 [5.7]	0%	Recruited through advertisement in the local media
1f	Fusar Poli et al. 2009	15 healthy men	26.67 [5.7]	0%	Recruited through advertisement in the local media
1 g	Fusar-Poli, P. et al. 2010	15 healthy men	26.67 [5.7]	0%	Recruited from advertisement in the local media
1 h	Winton-Brown, T. et al. 2011	14 healthy men	26.7 [5.7]	0%	Recruited advertisements in local media
2a	Wilson, R. et al. 2019	19 HC and 33 CHR	23.9 [4.15]	41%	HC recruited by local advertisement while CHR were recruited from early intervention services in the UK
2b	Davies. C 2022	19 HC and 33 CHR	23.4 [4.8]; 24.3 [4.73]	49%; 42%	HC recruited by local advertisement while CHR were recruited from early intervention services in the UK
2c	Bhattacharyya 2018	19 HC and 33 CHR	23.4 [4.8]; 24.3 [4.73]	49%; 42%	HC recruited by local advertisement while CHR were recruited from early intervention services in the UK
2d	Davies C 2020	19 HC and 33 CHR	23.4 [4.8]; 24.3 [4.73]	49%; 47.4%	HC recruited by local advertisement while CHR were recruited from early intervention services in the UK
3a	Lawn, W. et al. 2020	23 healthy participants	23.74 [4.2]	52%	Recruited through public advertisement
3b	Bloomfeild.M 2022	24 healthy participants	23.6 [4.12]	50%	Recruited through public advertisement
	fMRI: Resting State				
3c	Michael A P Bloomfield et al 2020	15 healthy participants	24 [5]	60%	Recruited through online adverts, posters and word-of-mouth
3d	Matthew B Wall 2022	23 healthy participants	23.8 [4.3]	52%	online adverts, posters and word-of-mouth
4	Grimm, O. et al. 2018	16 HC	NA	0%	Recruited via local advertisement
5a	Pretzsch, C. M. et al. 2019	17 neurotypicals, 13 ASD	28.47 [6.55]; 30.85 [9.79]	0%	na
	MRS				
5b	Pretzsch, C. M. et al. 2019	17 neurotypicals, 17 ASD	28.47 [6.55]; 31.29 [9.94]	0%	na
	PET				
6a	Crippa 2004	10 healthy volunteers	29.8 [5.1]	0%	Postgraduate students

*ASD* autism spectrum disorder, *CBD* cannabidiol, *HC* healthy controls

## Functional MRI (fMRI)

### Resting state fMRI

Four resting state fMRI studies (Bloomfield et al. 2020; Grimm et al. 2018; Pretzsch, Voinescu, et al. 2019; Wall et al. 2022) were identified. These studies examined brain activation by either measuring spontaneous low frequency fluctuations in BOLD signal while participants remain at rest in a MRI machine or cerebral blood flow (CBF) via the use of a technique called arterial spin labelling (ASL; (Barbier, Lamalle, & Décorps 2001)).

Firstly, in a double-blind, randomised, placebo-controlled (DBRCT) crossover study, 13 males with autism spectrum disorder (ASD) and 17 neurotypical males (mean [SD] age = 30.85 [9.79] years and 28.47 [6.55] years for ASD and neurotypical participants, respectively) received a single dose of 600 mg CBD or placebo (Pretzsch, Voinescu, et al., 2019). Participants were scanned 2 h after drug administration. CBD significantly increased spontaneous fluctuations in BOLD signal across both groups in the right fusiform gyrus (*p* = 0.041) and in the cerebellar vermis (*p* = 0.048) which post-hoc analyses demonstrated were driven by an effect in participants with ASD (*p*_FWE_ = 0.029 and *p*_FWE_ = 0.045; for fusiform and vermis clusters, respectively). Following a post-hoc seed-based analysis of functional connectivity in these regions of interest (ROIs), CBD was not shown to have a significant effect on vermal or fusiform functional connectivity with any other regions for neurotypical participants.

In another study (Grimm et al. 2018), 16 healthy male participants (demographic information not available) were included in a DBRCT crossover study with three separate arms and one-week intervals between scans. Participants were scanned 75 min following the consumption of either 10 mg THC, 600 mg CBD, or matched placebo. Seed-based analysis on four ROIs in the striatum, including the caudate (left and right) and putamen (left and right) were examined for connectivity with the rest of the brain. CBD administration led to an increase of fronto-striatal functional connectivity relative to placebo. Specifically, relative to placebo, those who were administered CBD showed a significant increase in connectivity between the right putamen seed (*p* < 0.03), and three clusters in the right prefrontal cortex (PFC). However, the analysis did not establish directionality.

In a crossover DBRCT, 15 participants (mean [SD] age = 24.1 [5.0] years, female = 60%) were administered either 600 mg CBD or placebo on separate days. The washout period was not reported, however, based on previous studies using the same sample, we can infer that the washout period was ≥ 1-week. Regional CBF was measured at rest 3 h after drug administration (Bloomfield et al. 2020). Compared to placebo, CBD administration significantly increased CBF in the hippocampus (15 mL/100 g/min [CI 5.78–24.21, *p* = 0.004]). CBF increased in the orbitofrontal cortex (*p* = 0.019) by 10.04 mL/100 g/min (CI, 1.90–18.19). However, only the effect in the hippocampus survived Bonferroni correction.

Finally, a crossover DBRCT was used to examine the effects of CBD on the striato-cortical connectivity of 23 healthy participants administered 600 mg CBD or a matched placebo (≥ 1-week washout period) 150 min before an MRI scan (mean [SD] age 23.8 [4.3] years, female = 53%)(Wall et al. 2022). CBD significantly decreased functional connectivity between subregions of the striatum, including limbic striatum activity with the lateral frontal cortex and the right hemisphere insula (*p* < 0.05), and between the sensorimotor striatum and cerebellum (*p* < 0.05). However, increased connectivity was observed between the associative striatum (regions receiving information from the associative areas of the cortex) and posterior parietal lobes (extending into the parieto-occipital sulcus and into the left posterior cingulate) (*p* < 0.05).

### Task based fMRI

Task-fMRI was the most common paradigm used to investigate the effect of CBD, with 14 of the 20 included studies employing task-fMRI. However, these 14 task fMRI studies comprised of data pertaining from three participant samples. In eight studies (Bhattacharyya et al. 2012; Bhattacharyya et al. 2014; Bhattacharyya et al. 2009; Bhattacharyya et al. 2010; S. J. Borgwardt et al. 2008; P. Fusar-Poli et al. 2010; Fusar-Poli et al. 2009; T. T. Winton-Brown et al. 2011), 15 male participants (mean [SD] age 26.7 [5.7]) were scanned using a crossover DBRCT, pseudo randomisation and a within group study design. Participants were either given THC 10 mg, CBD 600 mg or placebo 1 h prior to a task-fMRI scan with 1-month intervals between scanning sessions. Furthermore, four studies (Bhattacharyya, Wilson, Appiah-Kusi, O'Neill, et al., 2018; Davies et al. 2020; Wilson et al. 2019b) scanned one sample consisting of 19 healthy controls (HC) and 33 clinical high risk (CHR) for psychosis (mean [SD] age of 23.4 [4.8] and 24.3 [4.73]; 49% and 42% female, respectively). In these DBRCT parallel-arm studies, CHR participants were given 600 mg CBD or placebo, while HC did not receive any medication, 3 h prior to a scan. Finally, two more studies (Bloomfield et al. 2022; Lawn et al. 2020) examined 24 participants (mean [SD] age 23.6 [4.12], female = 50%), however, the study by Lawn et al. (2020) excluded one participant because they did not complete the MID task correctly (mean [SD] age 23.74 [4.2], female = 52%). In this crossover DBRCT, participants were given 600 mg oral dose of CBD or matched placebo and were scanned 150 min later with a 7-day washout period. Experimental tasks applied across all three participant samples included go/no-go (Bhattacharyya et al. 2010; S. J. Borgwardt et al. 2008), oddball tasks (Bhattacharyya et al. 2012, 2014), verbal paired memory (Bhattacharyya et al. 2009, 2010), fearful faces tasks (Bhattacharyya et al. 2010; Bloomfield et al. 2022; Davies et al. 2022; Davies et al. 2020; P. Fusar-Poli et al. 2010; Fusar-Poli et al. 2009), monetary incentive delay (Lawn et al. 2020; Wilson et al. 2019b), and passive visual and auditory presentations (T. T. Winton-Brown et al. 2011). The results are summarised by experimental task here.

#### Go/No go and oddball tasks

In go/no go tasks, participants are required to respond to appropriate, target “go” stimuli and not respond to inappropriate, “no-go” stimuli (Rubia et al. 2006). The number of false responses to “no-go” indicates inhibition capacity. Go/no-go tasks can be combined with oddball tasks to measure participants’ responses to novel stimuli, and ability to discriminate between salient or non-salient information. To do this, participants are presented with a series of repetitive stimuli that are irregularly interrupted by novel stimuli (the oddball stimulus) thereby providing information about how participants respond to novelty. S. J. Borgwardt et al., (2008) reported no significant drug effects on the combined go/no-go and oddball task performance, although there were different activation patterns on the ‘no-go’ relative to oddball trials between placebo and CBD conditions. Placebo administration revealed significant hyperactivation in the inferior and medial frontal gyri, the anterior insula, the anterior cingulate gyrus, and the supplementary motor area for ‘no-go’ compared to oddball condition (*p* < 0.0025). CBD administration showed hyperactivation in middle and superior temporal gyrus, insula, and posterior cingulate gyrus for ‘no-go’ compared to oddball condition (*p* < 0.0025). In comparison to placebo and for ‘no-go’ relative to oddball trials, CBD was associated with reduction in activity in the left insula and left superior and transverse temporal gyri (*p* < 0.01)*.* Bhattacharyya et al. (2012), in a secondary analysis of S. J. Borgwardt et al., (2008), reported results from a go/no-go task with added oddball stimuli to account for the novelty of ‘no-go’stimuli. Response latencies across all task conditions were significantly reduced in CBD groups compared to placebo (*p* = 0.01) with a trend towards higher reduction in response latency to oddball than standard stimuli (*p* > 0.01). During the task, CBD attenuated activation in clusters in the left medial PFC (*p* = 0.01) and augmented activation in clusters in the right caudate, parahippocampal gyrus, insula, precentral gyrus, and thalamus (*p* = 0.02), relative to placebo. In a follow-up analysis, seed clusters in the inferior frontal, dorsal, striatal and posterior hippocampal foci were selected as ROIs due to their involvement in processing deviant, rare or novel stimuli (Rubia, Smith, Brammer, & Taylor, 2007) and were shown to be functionally connected to multiple brain regions during the oddball task (Bhattacharyya et al. 2014). CBD attenuated functional connectivity from the inferior frontal gyrus seed cluster with a cluster with peaks in the left anterior lobe of the cerebellum, left thalamus, and lingual gyrus (*p* < 0.001) and attenuated functional connectivity with the right insula (*p* = 0.043). In the dorsal striatum seed cluster CBD augmented the functional connectivity of the left dorsal striatum with the body of the left caudate nucleus and the left inferior frontal gyrus (*p* = 0.008) and attenuated functional connectivity with the left anterior cingulate and the left medial frontal gyrus (*p* = 0.007). In the hippocampal seed cluster, functional connectivity of the left posterior hippocampal cluster with the left parahippocampal gyrus was augmented by CBD (*p* = 0.0045), whereas the functional connectivity between the right parahippocampal gyrus, the left posterior cingulate, and the tail of the left caudate was attenuated in the CBD condition (*p* = 0.004).

#### Verbal paired memory task

The verbal paired memory tasks used in the selected articles were adapted from the paired associate learning subtest of the Wechsler Memory Scale–Revised (Wechsler 1987). This task primarily assesses episodic memory and induces activity in various areas associated with memory. Bhattacharyya et al. (2009) investigated the impact of CBD on mediotemporal and PFC activation during a verbal paired association task. Performance on the task was not significantly affected by treatment. However, CBD administration did modulate regions associated with memory consolidation and including insula, mediotemporal gyrus, lingual gyrus, precuneus, and precentral gyrus activation during repeated encoding (*p* < 0.05) and the hippocampus during recall blocks relative to placebo (*p* = 0.01).

A similar study incorporating the verbal paired memory task by task (Bhattacharyya, Wilson, Appiah-Kusi, O'Neill, et al., 2018), in which CHR participants who received CBD demonstrated greater activation in the precentral gyrus compared to placebo, coupled with reduced activation in the parahippocampus extending to the superior temporal gyrus and cerebellum (*p* = 0.003) and precentral gyrus (*p* ≤ 0.003) during encoding phases. Additionally, CHR participants who received CBD showed greater activation than placebo in regions including the medial frontal gyrus, right precentral gyrus and adjacent cingulate gyrus, and the left cingulate gyrus and caudate body (*p* ≤ 0.002) during the recall phase of the task (Bhattacharyya, Wilson, Appiah-Kusi, O'Neill, et al., 2018). Generally, these activation patterns signified a trend towards the normalisation of activity in these regions and resembling activation patterns observed in HC.

#### Fearful faces

During the fearful faces task, images of faces that exhibit varying levels of fearful expressions are presented to the participants to elicit activity associated with emotional processing and anxiety responses (Keedwell, et al. 2005; Morris et al. 1996). Fusar-Poli et al. (2009) demonstrated that CBD reduced activity in the amygdala (*p* = 0.0012) and the anterior and posterior cingulate cortex (*p* = 0.00065 and *p* = 0.000432 respectively) while participants were processing intensely fearful faces. Moreover, CBD reduced activity in the posterior lobe of the cerebellum for moderately fearful face stimuli compared to placebo. Concurrently recorded electrodermal psychophysiological responses also demonstrated reduced skin conductance response (SCR) fluctuations for intensely fearful expression stimuli (*p* < 0.05) but not neutral or mildly fearful faces. This reduction of SCR fluctuations is a proxy for physiological arousal (Bach, Friston, & Dolan, 2010). The suppression of amygdala as well as the anterior cingulate covaried with the reductions in the number of SCR fluctuations (*r* = 0.524; *p* = 0.049) and, as reported in a later study (Bhattacharyya et al. 2010), a trend level anxiolytic effect as indexed by the State Trait Anxiety Inventory (*r* = 0.551, *p* = 0.017). Finally, the effect of CBD in modulating prefrontal-subcortical connectivity during emotion processing was investigated in a follow-up analysis (P. Fusar-Poli et al. 2010). CBD treatment led to significant disruption of forward connectivity between the amygdala and anterior cingulate observed in the placebo group while participants responded to fearful faces (*p* = 0.035).

In a DBRCT parallel arm study, the effect of CBD on both the mediotemporal and striatal function (Davies et al. 2020) was examined. Subsequently, the relationship between mediotemporal function and serum cortisol level during the fearful faces paradigm was examined in the same sample but using different techniques (Davies et al. 2022). During the processing of fearful faces, CHR participants in the placebo condition experienced greater activity in parahippocampal gyrus (*p* ≤ 0.003) and reduced activity in the striatum (*p* ≤ 0.002) compared to HC. Moreover, CHR participants receiving CBD, versus those who received placebo, showed hypoactivation in the parahippocampal gyrus and amygdala (*p* ≤ 0.002) and greater activation in the putamen (*p* ≤ 0.001). In the healthy control group, higher cortisol induced by social stress led to lower parahippocampal activation (*p* = 0.023)*.* CHR participants who received placebo showed a statistically significant difference between parahippocampal activation and cortisol when compared to controls who did not receive any treatment (*p* = 0.033). When CHR participants received CBD, they showed a similar relationship between cortisol and parahippocampal activation compared to healthy controls (*p* = 0.67). Conversely, Bloomfield et al (2022) demonstrated no significant drug effects on brain responses to emotional faces from any category (open-mouth happy/angry/neutral) when comparing CBD to placebo administered groups. However, this task did slightly differ from the previous face task as it used happy, fearful and neutral faces from the NimStim stimulus set (Tottenham et al. 2009).

#### Monetary incentive delay

Monetary Incentive Delay (MID) tasks present stimuli as cues that precede a monetary reward stimulus and can be used to measure the anticipation and feedback phases of reward processing (Knutson & Greer 2008). (Wilson et al. 2019a) demonstrated that CBD attenuated the observed hyper-activity in the left insula/parietal operculum in the CHR group which occurred during reward and loss anticipation stages of the task (*p* = 0.035) (Wilson et al. 2019). Additionally, (Lawn et al. 2020) revealed that a whole brain analysis resulted in insufficient statistical evidence to suggest that CBD modulated reward-related brain activity to a greater degree than placebo.

#### Passive listening and viewing of stimuli

Viewing and listening passively during an fMRI scan allows for the investigation of the neural correlates of visual and auditory processing (Brown et al. 2004). T. Winton-Brown et al. (2011) investigated the effect of CBD on visual (checkboards) and auditory processing (speech). During passive auditory processing, CBD increased activation in temporal cortex bilaterally extending medially to the insulae and caudally to the hippocampi and parrahipocampal gyri compared to placebo (*p* ≤ 0.007). During auditory processing, CBD also reduced activation in posterolateral parts of the left superior temporal gyri-incorporating portions of supramarginal gyrus, the insula, and posterior middle temporal gyrus (*p* = 0.002). During passive visual processing, CBD increased activation in the right occipital lobe, with the largest increases in the lingus gyrus, cuneus, and middle and inferior occipital gyrus (*p* = 0.0065). This study demonstrates that CBD modulates a variety of regions during passive visual and auditory processing.

## Magnetic Resonance Spectroscopy (MRS)

(Pretzsch, Freyberg, et al. 2019) investigated the effects of 600 mg of CBD on GABA and Glx (glutamine/glutamate) (N = 34 with ASD, mean [SD] age of 28.47 [6.55] years; N = 17 neurotypical controls) measured 2 h after drug administration. In the basal ganglia (BG), CBD increased Glx in both groups (*p*_uncorr_ = 0.070); in the DMPFC, CBD decreased Glx in both groups (*p*_uncorr_ = 0.055). There was a significant voxel × drug interaction effect (*p*_uncorr_ = 0.033) in both groups, CBD increased Glx in the BG and decreased Glx in the DMPFC (this effect did not survive Bonferroni-correction). CBD increased GABA + in the control group (surviving Bonferroni-correction (p_corr_ = 0.004)). This group × drug interaction was largely driven by changes in the DMPFC (*p*_uncorr_ = 0.038).

## SPECT

The search revealed only one study that had utilised SPECT methodology. Crippa et al. examined 400 mg of CBD versus placebo in a crossover DBRCT (*N* = 10, 7 day washout period) on resting blood flow using SPECT 110 min post-drug administration (J. A. Crippa et al. 2004). ROIs associated with limbic and paralimbic networks were selected a priori. Compared to placebo, CBD decreased uptake of a radiotracer contrast in clusters in the medio portion of the left amygdala-hippocampal complex and uncus extending into the hypothalamus and the superior section of the left posterior cingulate gyrus (*p* < 0.001). CBD also showed comparably increased activity in a cluster in the mediotemporal cortex including the left parahippocampal gyrus extending to include the left fusiform gyrus (*p* < 0.001). CBD was also associated with decreased subjective anxiety and increased mental sedation (*p* < 0.001) however there was no correlation between the mood scales and the ECD uptake (Table 1) .

### Quality assessment

Table 2 depicts the risk of bias as per each domain of the Cochrane RoB (Sterne et al. 2019). The randomisation processes for all studies were rated as having a *low risk of bias* (Domain 1). *Some concerns* were noted with respect to period and crossover effects (domain S) whereby some studies reported limited washout periods (e.g. 1-week ( Bloomfield et al. 2022; J. A. Crippa et al. 2004; Grimm et al. 2018; Lawn et al. 2020; Wall et al. 2022) or did not provide sufficient information regarding the washout period (Bloomfield et al. 2020). Although the half-life of CBD has previously been suggested to be up to 32 h (Ujváry & Hanuš, 2016) suggesting that 7 days may be a sufficient washout period, recent work showed that CBD has a long window of detection in plasma of up to 4 weeks post-drug administration (McCartney et al. 2022). No studies were considered to have risk of bias due to deviations from intended interventions (Domain 2) and there was low risk of bias due to missing data (Domain 3). Some potential concerns of bias in the outcomes that were measured (Domain 4) only were due to the potential of residual response due to test design. Concerns noted in the selection of reported results (Domain 5) were due to the majority of studies having no published pre-determined statistical analysis plan which may suggest potential vulnerability to selective analyses and reporting biases particularly relevant to fMRI data Table 3.

**Table 3:** Cochrane risk of bias

Reference	Domain 1; bias arising from the randomization process	Domain S; Risk of bias arising from period and carryover effects	Domain 2; bias due to deviations from the intended intervention	Domain 3; bias due to missing outcome data	Domain 4; bias in measurement of the outcome	Domain 5; bias in selection of the reported results	Overall risk of bias judgement
**Crossover**							
Bhattacharyya et al 2009	Low	Some concerns	Low	Low	Low	Some concerns	Some concerns
Bhattacharyya et al 2010	Low	Low	Low	Low	Low	Some concerns	Low
Bhattacharyya et al 2012	Low	Low	Low	Low	Some concerns	Some concerns	Low
Bhattacharyya et al. 2014	Low	Low	Low	Low	Low	Some concerns	Low
Bhattacharyya et al 2018	Low	Low	Low	Low	Low	Low	Low
Bloomfield et al 2020	Low	Some concerns	Low	Low	Low	Some concerns	Low
Bloomfield et al 2022	Low	Low	Low	Low	Low	Low	Low
Borgwardt et al 2008a, b	Low	Low	Low	Some concerns	Low	Some concerns	Some concerns
Crippa et al 2004	Low	Some concerns	Some concerns	Low	Low	Some concerns	Some concerns
Fusar Poli et al. 2009	Low	Low	Low	Some concerns	Low	Some concerns	Some concerns
Fusar-Poli et al 2010	Low	Low	Low	Low	Low	Some concerns	Some concerns
Grimm et al 2018	Low	Some concerns	Some concerns	Some concerns	Low	Some concerns	Some concerns
Lawn et al 2020	Low	Some concerns	Low	Some concerns	Low	Low	Some concerns
Pretszch et al. 2019	Low	Low	Low	Low	Low	Low	Low
Pretzsch 2019	Low	Low	Low	Low	Low	Low	Low
Wall et al 2022 (Study 1)	Low	Low	Low	Low	Low	Low	Low
Wall et al 2022 (Study 2)	Low	Low	Low	Low	Low	Low	Low
Winton-Brown et al 2011	Low	Low	Low	Some concerns	Low	Some concerns	Some concerns
Parallel							
Davies et al 2020	Low	Low	Low	Low	Low	Low	Low
Davies et al 2022	Low	Low	Low	Low	Low	Low	Some concerns
Wilson et al 2019	Low	Low	Low	Low	Low	Some concerns	Some concerns

## Discussion

This review synthesised neuroimaging literature examining the effects of CBD on neurobiology in healthy subjects and to further consider whether CBD may have promise in the management of AUD. We identified 20 neuroimaging studies that examined CBD in a healthy sample since 2004 which revealed broad modulatory effects across several brain regions and networks. Below we synthesise these results according to neuroimaging modality and then in light of converging neurobiological correlates associated with addictive behaviours.

Functional MRI was by far the most common neuroimaging modality accounting for 90% of the studies reviewed. Resting state fMRI was the focus of four studies presented in this review. Three studies demonstrated that CBD significantly modulated functional connectivity (Grimm et al. 2018) (Wall et al. 2022) and CBF (Bloomfield et al. 2020). CBD was shown to increase fronto-striatal coupling, from a seed in the right putamen to the PFC (Grimm et al. 2018); as well as increasing connectivity between “associative” striatum and parietal regions (Wall et al. 2022). Furthermore, CBD was observed to increase CBF to the hippocampus (Bloomfield et al. 2020). CBD was also demonstrated to produce minor decreases in functional connectivity in limbic and sensorimotor regions (Wall et al. 2022). However, one study showed non-significant differences between CBD and placebo on whole brain BOLD activity (Pretzsch, Voinescu, et al. 2019).Fourteen Task-based fMRI articles, published between 2008 – 2022, used task paradigms to examine reward processing, salience attribution, emotion regulation and executive functioning following CBD administration. During go/no-go and oddball tasks, which tests response inhibition and salience attribution, CBD was found to reduce activity in the left insula and left superior and transverse temporal gyri (S. J. Borgwardt et al. 2008). Further, while reducing response latencies, CBD was demonstrated to attenuate activation in left medial PFC and augment activation in right caudate, parahippocampal gyrus, insula, precentral gyrus and thalamus (Bhattacharyya et al. 2012). Increased fronto-striatal connectivity and reduced mediotemporal-prefrontal connectivity was also reported during attentional salience tasks following CBD administration (Bhattacharyya et al. 2014). During a learning and memory, verbal paired task, CBD was observed to modulate insula, midtemporal gyrus, lingual gyrus, precuneus, and precentral gyrus during repeated encoding phases and modulated hippocampus during recall. However, none of these results reached threshold for less than one false positive cluster (Bhattacharyya et al. 2009). During an emotional regulation and processing task, CBD administration led to a lower number of SCR fluctuations for intensely fearful stimuli, but not neutral or mildly fearful stimuli. This lower SCR covaried with reduced activity in the amygdala and anterior and posterior cingulate cortex (Fusar-Poli et al. 2009). Additionally, CBD was found to disrupt forward connectivity between the amygdala and anterior cingulate while participants responded to fearful faces (P. Fusar-Poli et al. 2010). This result is supported by another study by (Davies et al. 2022) whereby CBD administration decreased activation in the parahippocampal gyrus and amygdala and increased activation in the putamen during emotion processing in a CHR sample, and also normalised the relationship between cortisol and parahippcampal activation. This effect of CBD on brain activity during emotional processing was not replicated in a later study (Bloomfield et al. 2022), however, this study did not yield a significant task effect in response to neutral vs fear faces unlike previous studies which may explain the conflicting results. Functional MRI during MID tasks, which probe anticipation and feedback of reward processing, yielded mixed results. While CBD slowed reaction times in one study with attenuation of the hyperactivation of left insula/parietal operculum in a CHR sample (Wilson et al. 2019b), another study failed to observe any significance differences in whole brain modulation (Lawn et al. 2020).Only two other studies were identified by the search strategy, focusing on neurometabolie presence and cerebral blood flow. One study used MRS (Pretzsch, Freyberg, et al. 2019). This study demonstrated that CBD modulates primary inhibitory and excitatory neurometabolites by increasing the inhibitory neurotransmitter GABA + in BG and DMPFC while increasing the excitatory Glx (glutamate + glutamine) in the BG but decreasing in the DMPFC relative to placebo-treated individuals. Additionally, one SPECT imaging study satisfied the inclusion criteria to be included in this review (J. A. Crippa et al. 2004). These authors found that CBD decreased cerebral blood flow to clusters in the medio portion of the left amygdala-hippocampal complex and uncus extending into the hypothalamus and the superior section of the left posterior cingulate gyrus. CBD was also shown to increase activity in a cluster in the mediotemporal cortex including the left parahippocampal gyrus extending to include the left fusiform gyrus.

These results suggest that CBD may modulate certain neurobiological correlates of addictive behaviors. There is a well-researched link between chronic heavy alcohol use impairs reward processing, salience attribution, emotion regulation and executive functioning (including inhibition control, working memory and self-monitoring) through the perturbation of various brain networks implicated in the development and maintenance of AUD (Koob & Volkow 2016). Some of these networks include the mesocorticolimbic (MCL), salience, fronto-striatal, and the limbic networks (Koob & Volkow 2016). These networks rely on various neurotransmitter systems including dopamine, opioid, endocannabinoid, serotonin, GABA, and glutamate systems. It has previously been suggested that CBD may normalise this perturbed neurocircuitry and subsequently support positive behavioural changes (Fagundo et al. 2013). Here, neuroimaging findings support the notion that CBD may modulate neurocircuitry implicated in the maintenance of AUD.

Mesocorticolimbic and salience attribution networks, which are responsible for reward processing and salience attribution, are functionally and anatomically linked (McCutcheon et al. 2019). The cannabinoid 1 receptors (CB_1_R), of which CBD is a negative allosteric modulator (NAM), are commonly located on the presynaptic terminals of dopaminergic neurons (Fitzgerald et al. 2012; Laprairie et al. 2015). Therefore, CBD may normalise the increased reward and salience attribution to alcohol associate cues by down regulating dopaminergic signalling in both the MCL and salience network. Evidence of this can be seen through CBD’s effect on the insula which is a major junction for both the mesolimbic (McCutcheon et al. 2019) and salience networks (Goulden et al. 2014; Peyron, Laurent, & Garcia-Larrea, 2000; Seeley et al. 2007). Various studies presented in this review suggest that CBD may attenuate both insula activity (Wilson et al. 2019a); Bhattacharyya et al. 2012) and functional connectivity (Wall et al. 2022). Thus, CBD may act to normalise hyper-signalling in the insula found in those with AUD, reducing both salience attribution and the reward processing. Indeed, the insula has been shown to have a major role in interoception (Critchley 2004) and patients with lesions in the insula have been observed to show attenuated craving and abstinence from cigarettes (Naqvi et al. 2007). Furthermore, CBD was shown to modulate the hypothalamus, the amygdala, the thalamus, the anterior cingulate cortex, and the hippocampus which may be an indication that CBD may not only modulate the salience and reward processing but also emotion regulation.

Prolonged alcohol use can commonly lead to negative emotional states and impairment in limbic neurocircuitry and emotional processing (Jansen et al. 2019; Oscar-Berman & Marinković, 2007). Several studies have observed heightened activation in the amygdala in those with AUD relative to controls during fMRI affect reactivity tasks (Gilman et al. 2008; O'Daly et al. 2012). Across the studies included in this review, CBD induced modulation of the hippocampus during recall (Bhattacharyya et al. 2009); attenuation of the amygdala and ACC during fearful faces paradigms (Bhattacharyya et al. 2010; Bloomfield et al. 2022; Davies et al. 2022; Davies et al. 2020; P. Fusar-Poli et al. 2010; Fusar-Poli et al. 2009); decreased connectivity between the amygdala and the anterior cingulate during emotion processing (P. Fusar-Poli et al. 2010); normalisation of parahippocampal activity during encoding processes (Bhattacharyya, Wilson, Appiah-Kusi, O'Neill, et al. 2018) and fear processing (Davies et al. 2020); and the relationship between cortisol and parahippocampal activity in CHR participants during fear processing (Davies et al. 2022). These results support the idea that CBD administration demonstrates interactions with limbic, particularly amygdala and ACC, activity as well as the functional connectivity between the amygdala and ACC. This modulation of the limbic network may be due to a number of mechanisms such as NAM action on CB_1_Rs (Campos & Guimarães 2008; Russo et al. 2005), alterations 5-HT1A in the amygdala and hippocampus and/or the release of pro-opiomelanocortin, corticotropin-releasing factor and glucocorticoid receptor gene expression following acute stress exposure (Viudez-Martínez, García-Gutiérrez, & Manzanares 2018).

Finally, CBD may induce improvements in reward processing, salience attribution and emotion regulation due to top-down control through increased fronto-striatal functional connectivity. In this review, several studies demonstrated increased fronto-striatal connectivity following CBD administration (Bhattacharyya et al. 2014; Grimm et al. 2018) (Wall et al. 2022) and therefore, improved executive functioning. In the context of AUD, deficits in executive functioning have been thought to be due to deficits of GABAergic signalling from the PFC (George et al. 2012). Further, glutamatergic projections from the PFC to the VTA in rats controls dopaminergic activity in the mesocortical pathway (Geisler & Wise 2008). This excitatory signalling to the VTA has been suggested to be involved in increasing conditioned behaviour and incentive salience in the presence of alcohol related cues (Lapish, Seamans, Judson Chandler, & Research 2006). To this degree, one study demonstrated CBD to increase GABAergic but decrease glutaminergic signalling from the DMPFC (Pretzsch, Freyberg, et al. 2019) which may therefore be relevant to alcohol recovery by improving both executive functioning and reducing cue induced craving and conditioned alcohol-seeking behaviour.This review identified several limitations in the studies that have utilised neuroimaging methods to examine the effect of CBD on the brain. Firstly, with regards to fMRI studies, there was a lack of consistency of imaging tasks and substantial methodological heterogeneity across the studies which therefore limit conclusions regarding CBD-induced neurobiological modulations to be relatively task specific. In addition, the 20 studies found in our search were obtained from only 6 different participant samples following completion of long neuroimaging protocols (see the outer ring of the sunburst plot, See Appendix Fig. 2). Thus, it is possible that the literature base may be subject to some bias due to sample specific effects and limited heterogeneity. Comprehensive and longer neuroimaging protocols may be vulnerable to task fatigue (Wylie, et al. 2020) and poorer data collection due to movement artifacts and scanner drift (Kopel et al. 2019). Moreover, there was diversity with regards to the timing between drug administration, scan time and also washout periods between sessions in crossover studies. In addition, the lack of pre-published protocols may lead to selective analyses which may be particularly relevant for fMRI studies. Additionally, as a meta-analysis could not be conducted due to the number of outcome variables, there may be involuntary bias in reporting results which were unintentionally favoured. Finally, while this review provides evidence for CBD’s modulation of neurocircuitry implicated in AUD-related behaviours, certain results suggest some non-significant results (Bloomfield et al. 2022; Lawn et al. 2020) and some are conflicting (Bloomfield et al. 2020; J. A. Crippa et al. 2004). Further, as results presented here may not translate to effects in AUD clinical profiles, directly examining the effect of CBD in AUD participants is required before determining the mechanisms by which CBD may function as a therapeutic use in this population. Recommendations for future research include publication of protocols to reduce deviation from protocol bias and selective analyses, optimised study design to reduce participant fatigue, ensuring a sufficiently long washout period between the crossover sessions, and consistent drug-scan administration time relative to peak plasma CBD concentrations.

In conclusion, previous research suggests that CBD may affect salience, reward, emotion generation and regulation and executive control (including inhibition control, working memory and self-monitoring) processes. These processes are highly relevant to alcohol seeking behaviours, suggesting that CBD may have potential in the management of alcohol use disorder. Although not supported by all the studies presented, the majority of the neuroimaging literature presented in this systematic review suggests that CBD may normalise these processes through its effect on mesocorticolimbic, limbic, salience and fronto-striatal signalling. Various limitations may explain some of the discrepancy in results including heterogenous methodological designs, the same or similar participant samples being used across different studies, variable drug administration times, possible carryover effects and participant fatigue due to long imaging protocols. Given the relevance of the networks affected by CBD in this review in alcohol seeking behaviour and relapse, research into the effect of CBD on brain and behaviour in populations with AUD to determine any potential role for management is warranted.

## Data Availability

Not applicable.

## Appendix

**Search terms used to search on** 06/10/22.

## EMBASE (253):

(MRS or Magnetic Resonance Spectroscopy or Spectroscopy or Metabolite Concentrations or magnetic resonance spectroscopy or MRS or functional magnetic resonance imaging or fMRI or resting state functional or magnetic resonance imaging or rsfMRI or structural magnetic resonance imaging or MRI or magnetic resonance spectroscopy or PET or positron emission tomography).mp. and (cannabidiol.mp. or (exp cannabidiol/ or exp cannabidiol derivative/)) [mp = title, abstract, heading word, drug trade name, original title, device manufacturer, drug manufacturer, device trade name, keyword, floating subheading word, candidate term word].

## PUBMED (228):

Search: (MRS OR Magnetic Resonance Spectroscopy OR Spectroscopy OR Metabolite Concentrations OR magnetic resonance spectroscopy OR MRS OR functional magnetic resonance imaging OR fMRI OR resting state functional OR magnetic resonance imaging OR rsfMRI OR structural magnetic resonance imaging OR MRI OR magnetic resonance spectroscopy OR PET OR positron emission tomography) AND (cannabidiol).

## PSYCINFO (185):

(MRS or Magnetic Resonance Spectroscopy or Spectroscopy or Metabolite Concentrations or magnetic resonance spectroscopy or MRS or functional magnetic resonance imaging or fMRI or resting state functional or magnetic resonance imaging or rsfMRI or structural magnetic resonance imaging or MRI or magnetic resonance spectroscopy or PET or positron emission tomography).mp. and (exp Cannabinoids/ or cannabidiol.mp.) [mp = title, abstract, heading word, table of contents, key concepts, original title, tests & measures, mesh].

## Medline (101):

(MRS or Magnetic Resonance Spectroscopy or Spectroscopy or Metabolite Concentrations or magnetic resonance spectroscopy or MRS or functional magnetic resonance imaging or fMRI or resting state functional or magnetic resonance imaging or rsfMRI or structural magnetic resonance imaging or MRI or magnetic resonance spectroscopy or PET or positron emission tomography).mp. and (cannabidiol.mp. or exp Cannabidiol/).

[mp = title, abstract, original title, name of substance word, subject heading word, floating sub-heading word, keyword heading word, organism supplementary concept word, protocol supplementary concept word, rare disease supplementary concept word, unique identifier, synonyms].
